# Elements of Surgery

**Published:** 1842-12

**Authors:** 


### Bibliographical Notices.

---

Art. IV.—*Elements of Surgery.* By Robert Liston, Surgeon to the North-London Hospital, Professor of Clinical Surgery, &c , &c. From the second London edition, *with copious notes and additions*, by Samuel D. Gross, M. D., Professor of Surgery in the Louisville Medical Institute, Surgeon to the Louisville Marine Hospital. Illustrated with numerous engravings. Philadelphia: Ed. Barrington and Geo. D Haswell; Louisville, James Maxwell, Jr. 1842. pp. 640, 8vo.

We do not know any work that has been received with such general commendation as this of Mr. Liston. The first edition rendered the author's name familiar to the profession in this country as that of a most dextrous and skilful surgeon, and an acute and original thinker. On its first appearance, it took rank immediately as one of the most valuable treatises in the language, and every year has but served to render it still more popular. It has proved equally acceptable to the juniors and the elders of the profession. To the former it particularly commends itself by the general perspicuity and vivacity of style, the plain, straightforward mode in which every thing is discussed, the simplicity of the rules of treatment and the clearness with which they are laid down: to the latter, by the useful hints it contains, and the boldness and originality of many of the author's opinions.

To give an analysis of a work so well known, would be useless, even if our space warranted it. It is sufficient to say that the present edition, having been carefully revised by the distinguished author prior to its publication abroad, is improved or changed so far as it may be supposed the experience of several years spent in the constant practice and cultivation of surgery, would extend his knowledge or modify his views. Nevertheless, as it came from the English press it was still imperfect, and the labors of the American editor are intended to supply the deficiencies and omissions apparent in the English edition.

The notes and additions of Dr. Gross amount to about seventy-five pages in all. Among the latter we notice articles on strabismus, club-foot, hydrocele or encysted tumor of the neck, hydatic tumor of the bone, &c. The notes are numerous, dispersed throughout the volume, and will be found to embody a mass of valuable information. We cannot forbear making a few quotations from this portion of the work, short as is the space allotted to us.

In the article on club-foot, Dr. Gross is disposed to adopt the views of Guerin of Paris, who "supposes that the primary mischief is in the nervous system, and that the spasmodic and permanent shortening of the muscles of the affected limb, is altogether consecutive." He deprecates the indiscriminate use of the knife for the cure of this deformity, and thinks that a majority of cases occurring in young subjects may be relieved without a resort to cutting.

"It is still a disputed point, whether, in the treatment of this affection, particularly in infants and young subjects, it is necessary or even justifiable, to divide, as a preliminary step, the tendons of the muscles which are instrumental in keeping up the distortion. Without endeavoring to settle this question, for which the time has not perhaps yet arrived, I must express my conviction that the present rage for tenotomy is calculated to do a vast deal of harm, not only in individual cases. many of which do not require it, but, what is worse and more deeply to be lamented, in bringing discredit upon an operation, which, if judiciously performed, cannot fail to be of the greatest benefit. In most of the cases occurring in children under two or three years of age, division of the tendons is altogether unnecessary; indeed, one of our most distinguished orthopedic surgeons, Dr. Chase of Philadelphia, seems to trust almost entirely to the employment of apparatus, and to resort to tenotomy only in the worst grades of the disease."

In the treatment of hydrocele, of the several varieties of which the text gives a very good account, Mr. L. prefers the method by injection, using for this purpose the pure port wine. The editor, on the contrary, objects to this method, as being not always successful, and sometimes requiring frequent repetition before a sufficient amount of adhesive action is obtained to obliterate the vaginal sac.

"Moreover, by carelessness on the part of the surgeon, the canula may slip out of the vaginal sac, and so allow the fluid to pass into the cellular substance of the scrotum, where, if it be not speedily evacuated by free incisions, it is sure to occasion gangrene. But this is not all. The operation, even when well performed, is sometimes followed by violent inflammation and suppuration; in one instance, indeed, I knew it to be productive of tetanus."

These considerations, he goes on to say, should be sufficient to deter the practitioner from resorting to this remedy, especially when it is remembered that we have another, not only entirely devoid of danger but always successful.

"This remedy is the seton, which I have been in the habit of employing, in repeated instances, for some years past, and from which I have never experienced any other than the most happy results. The operation is perfectly simple, the amount of inflammation produced by the presence of the foreign body may be easily regulated, and there is no danger of sloughing of the scrotum, much less of the developement of tetanus, or other mischief."

Certainly, so simple a proceeding is far better than the paraphernalia of elastic bottles and brass stop-cocks. The same means is recommended by Dr. Gross in the radical treatment of what has been denominated hydrocele of the neck, an encysted tumor occurring in that region, and met with in both sexes and at various periods of life.

One of the most interesting and valuable notes appended to the work, is that on wounds of the intestines, accompanying penetrating wounds of the abdomen. The directions given for their management by Dr. Gross, possess peculiar claims to our confidence from the fact that they are founded on the results of original investigations pursued by him during the past summer. These results entire, constitute a highly valuable paper, which we shall have the pleasure of presenting to our readers in the next volume of the journal. According to Dr. Gross, the grand principle which should guide the surgeon in these cases, is to close the opening so completely as to guard against the effusion of fecal matter. This done, the patient is comparatively safe, or free from the danger of peritoneal inflammation.

"To attain this object, the continued, or glover's suture as it is termed, is unquestionably preferable to any other, especially when made, as I would suggest it should be, with a small sewing-needle, armed with fine silk, and passed between the muscular and mucous coats, or, what is the same thing, through the substance of the cellulo-fibrous lamella. After the suture has been applied, the protruded part of the mucous lining, if there be any, should be pared off with a sharp knife, to facilitate the process of reparation, the surface of the bowel should be cleansed with tepid water, and the whole carefully returned into the abdomen. If the interrupted suture be used, the interval between each two respective threads must not exceed two lines, or the sixth of an inch, otherwise there will be danger of fecal extravasation, and the ends, instead of being brought out at the external aperture, should be cut off close to the knots. The reason why I prefer the continued suture, made in the manner above mentioned, is simply because we can thereby more effectually close the wound, at the same time that the parts are placed in the best possible condition for speedy reunion, from the want of protrusion of the lining membrane, and consequently the more perfect contact of the serous surfaces.

The ligatures which are employed in sewing up a wounded intestine are detached at a period varying from ten days to three or four weeks, according to the nature of the suture. When the extremities are cut off close to the knots, they invariably fall into the cavity of the bowel, and are finally discharged along with the feces; if, on the other hand, they are brought out at the external opening, they pass off in that direction instead of the one just mentioned."

If the opening in the gut does not exceed three or four lines in extent, Dr. G. recommends the plan of Cooper, to encircle it with a ligature. The latter should be drawn pretty firmly to prevent slipping, and the ends cut off close to the knot. It generally makes its way into the bowel in eight or ten days.

"When the bowel is completely severed, or mortified in its entire calibre, the edges, after being properly prepared, should be brought in contact, and retained by the continued or the interrupted suture. Cases of this kind, although apparently desperate, are not always of so hopeless a character as might at first sight be supposed. This is shown, not only by experiments on the inferior animals, but by what occurs in the human subject, in sphacelated hernia, and in intus-susception. In the former, the greater part, or even the whole, of the circumference of the tube may be destroyed, and yet the patient ultimately recover, with perhaps the temporary inconvenience merely of an artificial anus; and in the latter, large pieces are not unfrequently detached without any serious suffering, save what is experienced during the antecedent and concomitant inflammation. In my morbid collection is a preparation of this kind, evidently a portion of the colon, nearly a foot long, which was discharged by a child six years old, who, notwithstanding, made a speedy and perfect recovery. Thirty-five cases of a similar character, collected from the writings of different pathologists, have been reported by Dr. Thompson of Europe. In a dog, from which I removed two inches and a half of the ileum, and treated the edges of the wound with six interrupted sutures, complete recovery took place, unattended with a single bad symptom. The threads were introduced at equal distances from each other, with a small sewing-needle, and the ends cut off close to the knots. Four months after the operation, being in good health, and the outer wound entirely healed, he was killed. Externally the bowel was perfectly smooth and natural, as if no injury had ever been inflicted upon it: the mucous membrane was of the same appearance as elsewhere, with the exception of a small depression corresponding with the edges of the wound."

These quotations, which we have taken at random, without any reference even to the order in which they occur, will perhaps suffice to give the reader an idea of the repast that awaits him in the book itself.

We must not forget to mention that the volume is rendered still more attractive by the addition of numerous wood engravings (some of them introduced by Dr. Gross), all finely executed. These will be found of very considerable advantage to the student, materially assisting him in comprehending the explanation of morbid structure. Another admirable feature, is the printing of the notes in type of the same size as that of the text. This obviates almost entirely, whatever objections can be alleged against foot-notes. C.

